# Ancient Origin and Gene Mosaicism of the Progenitor of *Mycobacterium* *tuberculosis*

**DOI:** 10.1371/journal.ppat.0010005

**Published:** 2005-08-19

**Authors:** M. Cristina Gutierrez, Sylvain Brisse, Roland Brosch, Michel Fabre, Bahia Omaïs, Magali Marmiesse, Philip Supply, Veronique Vincent

**Affiliations:** 1 Laboratoire de Référence des Mycobactéries, Institut Pasteur, Paris, France; 2 Unité de Biodiversité des Bactéries Pathogènes Emergentes, Institut Pasteur, Paris, France; 3 Unité de Génétique Moléculaire Bactérienne, Institut Pasteur, Paris, France; 4 Laboratoire de Biologie Clinique, HIA Percy, Clamart, France; 5 INSERM U629, Institut Pasteur de Lille, Lille, France; University of Washington, United States of America

## Abstract

The highly successful human pathogen *Mycobacterium tuberculosis* has an extremely low level of genetic variation, which suggests that the entire population resulted from clonal expansion following an evolutionary bottleneck around 35,000 y ago. Here, we show that this population constitutes just the visible tip of a much broader progenitor species, whose extant representatives are human isolates of tubercle bacilli from East Africa. In these isolates, we detected incongruence among gene phylogenies as well as mosaic gene sequences, whose individual elements are retrieved in classical *M. tuberculosis*. Therefore, despite its apparent homogeneity, the *M. tuberculosis* genome appears to be a composite assembly resulting from horizontal gene transfer events predating clonal expansion. The amount of synonymous nucleotide variation in housekeeping genes suggests that tubercle bacilli were contemporaneous with early hominids in East Africa, and have thus been coevolving with their human host much longer than previously thought. These results open novel perspectives for unraveling the molecular bases of *M. tuberculosis* evolutionary success.

## Synopsis

*Mycobacterium tuberculosis,* the agent of tuberculosis, is a highly successful human pathogen and kills nearly 3 million persons each year. This pathogen and its close relatives sum up in a single and compact clonal group dating back only a few tens of thousands of years. Using genetic data, the researchers have discovered that human tubercle bacilli from East Africa represent extant bacteria of a much broader progenitor species from which the *M. tuberculosis* clonal group evolved. They estimate that this progenitor species is as old as 3 million years. This suggests that our remote hominid ancestors may well have already suffered from tuberculosis. In addition, the researchers show that tubercle bacilli are able to exchange parts of their genome with other strains, a process that is known to play a crucial role in adaptation of pathogens to their hosts. Thus, the *M. tuberculosis* genome appears to be a composite assembly, resulting from ancient horizontal DNA exchanges before its clonal expansion. These findings open novel perspectives for unraveling the origin and the molecular bases of *M. tuberculosis* evolutionary success, and lead to reconsideration of the impact of tuberculosis on human natural selection.

## Introduction

Most bacterial species consist of a wide spectrum of distinct clones or clonal complexes [1–3] that differ from one another by 1% or more at synonymous nucleotide sites [4,5]. Intraspecies genetic diversity is usually generated both by mutations and by horizontal genetic exchanges. However, some important human pathogens such as *Salmonella enterica* serotype Typhi [6] and *Yersinia pestis* [1] essentially consist of a single specialized clone that recently evolved from a well-known more diversified progenitor species. Members of the *Mycobacterium tuberculosis* complex (MTBC), the agents responsible for tuberculosis, are among the most successful human pathogens. The MTBC as defined here comprises the so-called *M. tuberculosis, M. bovis, M. microti, M. africanum, M. pinnipedii,* and *M. caprae* species. Although the members of the MTBC display different phenotypic characteristics and mammalian host ranges, they represent one of the most extreme examples of genetic homogeneity, with about 0.01%–0.03% synonymous nucleotide variation [7–12] and no significant trace of genetic exchange among them [8,13–15]. Therefore, it is believed that the members of the MTBC are the clonal progeny of a single successful ancestor, resulting from a recent evolutionary bottleneck that occurred 20,000 to 35,000 y ago [7,8,11,16].

However, the nature and the boundaries of the bacterial pool that existed prior to the putative bottleneck, as well as the time of the transition to pathogenicity for mammalian hosts, have not yet been identified. A preliminary report suggested that *M. canettii,* a rare tubercle bacillus with an unusual smooth colony phenotype [17], could represent the most ancestral lineage of the MTBC [18]. However, this speculation relied only on the identification of one to four nucleotide polymorphisms in a single gene. Here, based on an extensive genetic analysis including seven genes, we found that *M. canettii* and other smooth tubercle bacilli actually correspond to pre-bottleneck lineages, belonging to a much broader progenitor species from which the MTBC emerged.

## Results/Discussion

### Identification of Clonal Groups of Smooth Tubercle Bacilli

We extensively characterized 37 pulmonary and extra-pulmonary isolates of smooth tubercle bacilli (see Material and Methods; Table S1) from European and African patients, mostly immunocompetent subjects who live or have lived in Djibouti, East Africa. Genotyping with a broad set of repetitive DNA and long sequence polymorphism markers led to recognition of eight clonal groups, designated A to I, within which the markers were virtually identical (Figure S1; Table S2). According to these markers, only groups A and C/D corresponded to *M. canettii* isolates, as defined by van Soolingen et al. [17] and Brosch et al. [16]. Group B was closely related to *M. canettii* but differed by the presence of *RD12^can^,* characteristically deleted in *M. canettii,* and by the absence of IS*1081* insertion sequence. The five other groups of smooth tubercle bacilli were remarkably distinctive from *M. canettii* and the thousands of MTBC strains globally investigated up to now, notably by lacking IS*1081* and/or the direct repeat (DR) locus [19].

### Smooth Tubercle Bacilli and MTBC Form a Single Mycobacterial Species

**Figure 1:**
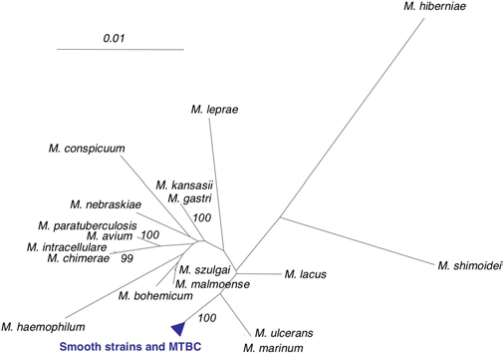
Phylogenetic Position of the Tubercle Bacilli within the Genus *Mycobacterium* The blue triangle corresponds to tubercle bacilli sequences that are identical or differing by a single nucleotide. The sequences of the genus *Mycobacterium* that matched most closely to those of *M. tuberculosis* were retrieved from the BIBI database (http://pbil.univ-lyon.fr/bibi/) and aligned with those obtained for 17 smooth and MTBC strains. The unrooted neighbor-joining tree is based on 1,325 aligned nucleotide positions of the 16S rRNA gene. The scale gives the pairwise distances after Jukes-Cantor correction. Bootstrap support values higher than 90% are indicated at the nodes.

To determine the positions of the smooth tubercle bacilli within the *Mycobacterium* genus, we classically sequenced portions of six housekeeping genes *(katG, gyrB, gyrA, rpoB,* *hsp65,* and *sodA)* and the complete 16S rRNA gene of all isolates of groups A, B, E, F, G, H, and I, of representative isolates of group C/D, and of representative strains of the MTBC members (Table S3). Consistent with the analysis involving repetitive DNA and long sequence polymorphism markers, all gene fragments were identical for smooth strains belonging to the same group, but differed between the groups. The comparison of the sequences of 16S rRNA (Figure 1) and these housekeeping genes (data not shown) with those of other mycobacterial species demonstrated that the eight groups of the smooth strains and MTBC members form a single species, defined by a compact phylogenetic clade remote from the other species of the *Mycobacterium* genus. The 1,537-bp 16S rRNA sequences of smooth groups E to I were identical to their MTBC counterparts, whereas the sequences of groups A to D differed only by a single nucleotide from the MTBC.

### Population Structure of the Tubercle Bacilli Species

The DNA sequences of multiple housekeeping genes can be used to infer the population structure and the phylogenetic history of bacterial species [1–4]. To investigate the population structure of the tubercle bacilli species, we aligned the 3,387 nucleotides sequenced in the six housekeeping genes of the representative smooth and MTBC isolates. The alignment revealed no insertions or deletions. We identified 52 polymorphic nucleotide sites (1.54%), of which 46 were synonymous substitutions. Two of the six nonsynonymous sites were located in the *katG* and *gyrA* genes. These two mutations, together with the presence of the *TbD1* and *RD9* genomic regions in all the smooth isolates, classify the smooth strains among the most ancient phylogenetic lineages of tubercle bacilli [7,16].

**Figure 2:**
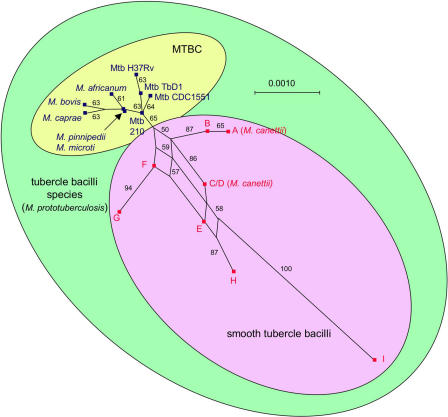
Splits Graph of the 17 Concatenated Sequences of the Six Housekeeping Genes The nodes represent strains and are depicted as small red (smooth tubercle bacilli) or blue (MTBC members) squares. The scale bar represents Hamming distance. Numbers at the edges represent the percent bootstrap support of the splits obtained after 1,000 replicates. The fit was 61.7%. Note that the branching order of MTBC strains is weakly supported, and it should therefore not be seen as contradicting previous evolutionary hypotheses based on deletion patterns [16].

Each unique gene sequence was assigned a different allele number, resulting in two to 11 alleles per gene. The distances between the various alleles were calculated using the mean percent divergence at synonymous (Ks) and nonsynonymous sites (Ka). The distances between the alleles of the MTBC strains were always much smaller than those between the alleles of the smooth strains (Table 1). Furthermore, the distances between the MTBC alleles and the smooth tubercle bacilli alleles were within the range observed in the smooth strains alone, with the minor exception of *hsp65.* These results show that the whole MTBC is only a subset of the larger tubercle bacillus species defined by the smooth groups. Consistently, phylogenetic analysis using a split decomposition graph showed that the MTBC forms a single compact bifurcating branch, rooted within the much larger array constituted by the smooth groups (Figure 2).

**Table 1:**
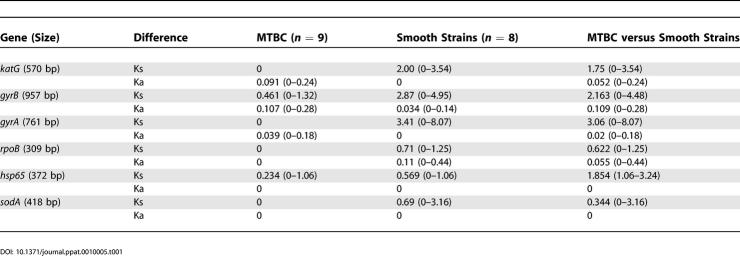
Mean Percent Pairwise Differences at Synonymous (Ks) and Nonsynonymous (Ka) Sites

DOI: 10.1371/journal.ppat.0010005.t001

The mean synonymous distance among distinct alleles in the tubercle bacilli (0.0083–0.039) was similar to that observed in many bacterial species known to be diverse, such as *Staphylococcus aureus* (0.023–0.037) [4,5,20]. Most of the synonymous nucleotide substitutions were found only in the smooth tubercle bacilli (41/46). Our fluctuation tests [21] showed that the frequency of spontaneous drug resistance mutations in the smooth and the MTBC bacilli was similar (data not shown), arguing against the possibility that the observed nucleotide diversity of the smooth bacilli is caused by hypermutation. Likewise, the ratio of synonymous to nonsynonymous substitutions of the smooth tubercle bacilli (Ks/Ka = 33.3) is close to values observed in other bacteria (ranging from 7.2 to 39.6) [22,23], but much higher than the value of 1.6 found when comparing the whole genomes of *M. tuberculosis* CDC1551 and H37Rv strains [10]. This high Ks/Ka value is consistent with purifying selection acting against amino acid changes over long time periods, leading to relative accumulation of synonymous versus nonsynonymous mutations. In contrast, the low Ks/Ka value observed within the MTBC is consistent with recent expansion [4,10].

These results demonstrate that, similar to *Y. pestis* or *S. enterica* serotype Typhi [1,6], the MTBC consists of a successful clonal population that recently emerged from a much more ancient and large bacterial species, engulfing *M. canettii* and the other smooth groups. This supports the bottleneck hypothesis [7,16]. We propose to name this species *M. prototuberculosis,* to reflect its status as the *M. tuberculosis* progenitor (Figure 2).

### Gene Mosaicism of Tubercle Bacilli

**Figure 3:**
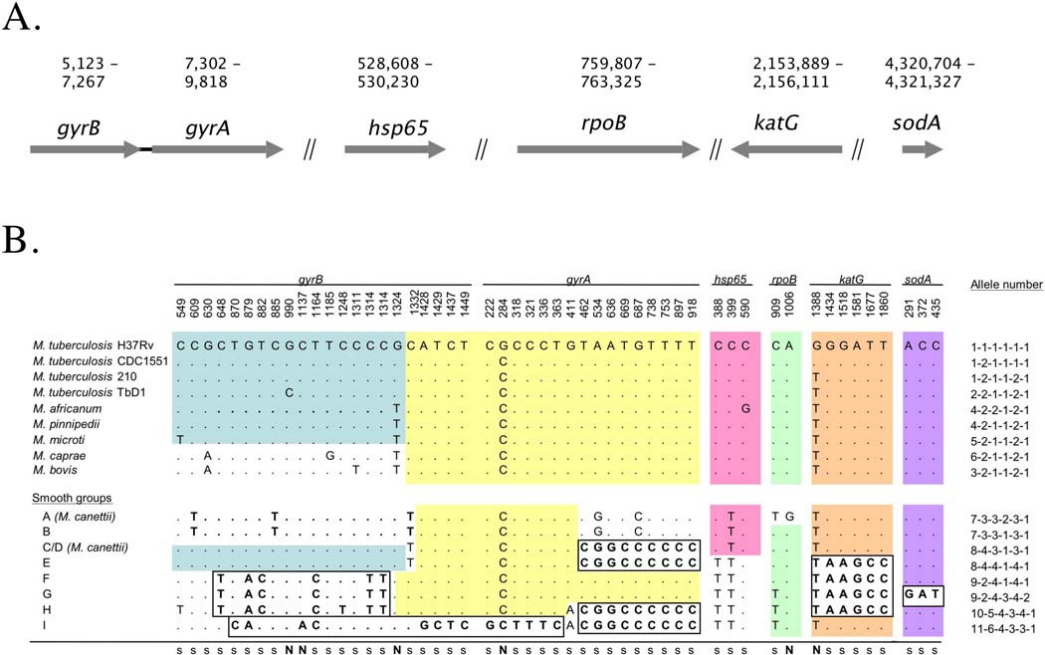
Nucleotide Polymorphism Detected in the Six Housekeeping Genes for the 17 Sequenced Strains (A) Location of the genes on the genome of *M. tuberculosis* H37Rv. Note that *gyrB* and *gyrA* are adjacent. (B) Pattern of polymorphic sites revealing mosaicism of sequences. Colored blocks correspond to sequence stretches in the smooth strains that are similar or identical to the sequences in the MTBC. Boxes correspond to blocks of consecutive nucleotides in smooth strains that differ by at least three nucleotides from *M. tuberculosis* H37Rv. The last column indicates the allele number for each gene. Letters N and s indicate nonsynonymous and synonymous substitutions, respectively.

To investigate the contribution of horizontal DNA exchanges to the genetic diversity of *M. prototuberculosis*, we investigated split decomposition of concatenated sequences [24] and the congruence of individual gene phylogenies [25]. The network structure linking the smooth strains in the splits graph (Figure 2) revealed incongruence between their gene sequences. We also found strong inconsistencies among phylogenies of individual gene sequences (Figure S2). Furthermore, the detection of several sequence mosaics in the *gyrB* and *gyrA* gene sequences provided direct evidence of intragenic recombination among the smooth strains (see boxes in Figure 3). These two genes form a single operon. As an example of mosaics, the *gyrB* and *gyrA* sequences of smooth groups C/D and E are composed of two large blocks separated by *gyrA* position 461. One of these blocks is almost identical to the sequence of *M. tuberculosis* and the other is identical to the sequence in groups H and I. The significance of sequence mosaicism was supported by maximum chi-square (*p* < 0.005) and Sawyer's (*p* < 0.05) statistical tests. In contrast, the rare minor allele differences among the smooth strains, such as those between *gyrB* alleles 9 and 10, are probably due to point mutations rather than recombination. Altogether, these observations provide evidence that both mutations and DNA recombination have occurred during the evolution of smooth tubercle bacilli.

In contrast, using the same analysis, no evidence of recombination was detected among the MTBC strains, consistent with their previously reported clonal population structure [13–15]. Remarkably, however, when compared to *M. prototuberculosis,* the concatenated sequences of the six housekeeping genes of the MTBC strains appear to be constituted of a mosaic of patches identical or nearly identical to sequence patches from different smooth groups (see colored blocks in Figure 3). This sequence patchwork suggests that the chromosomal framework of the MTBC, despite its present clonal and highly conserved structure, is actually a composite assembly of genetic sequences resulting from multiple remote horizontal gene transfer events. These DNA transfer events likely took place in the pool of the progenitor tubercle bacilli before the expansion of the MTBC clone. Therefore, the apparent absence of recombination among the MTBC strains after the bottleneck could have several potential explanations: the MTBC strains could have lost the capacity of horizontal gene transfer, horizontal gene transfer events are too rare among tubercle bacilli to have occurred since the MTBC bottleneck, or the MTBC ecological niche differs from that of *M. prototuberculosis* and offers no opportunity for recombination events.

### Ancient Origin of the Tubercle Bacilli Species

Synonymous nucleotide diversity can be used to estimate the minimal age of the last common ancestor of a species [22,23]. The average pairwise difference at synonymous sites (Ks) across the six housekeeping genes for the 17 sequenced strains was 0.0148 (Protocol S1). Given previous studies that estimated the age of *M. tuberculosis* to be approximately 35,000 y based on bacterial synonymous substitution rates of 0.0044–0.0047 per site per million years [11,26,27], we estimated that the minimal time needed to accumulate the observed amount of synonymous divergence in the tubercle bacilli species was between 2.6 and 2.8 million y. As both smooth bacilli and *M. tuberculosis* are isolated from human tuberculosis cases, the most parsimonious hypothesis is that the last common ancestor of the tubercle bacilli species could already have caused human tuberculosis. Therefore, our results change the current paradigm of the recent origin of tuberculosis [7] by suggesting that its causative agent is as old as 3 million years. Tuberculosis could thus be much older than the plague [1], typhoid fever [6], or malaria [28], and might have already affected early hominids. Consistent with this speculative scenario, nearly all smooth tubercle bacilli isolated so far come from East Africa, a region where early hominids were present 3 million years ago [29]. The distribution of diversity between the variable smooth tubercle bacilli from Djibouti and the uniform worldwide MTBC is remarkably reminiscent of the distribution of human genetic diversity among world populations, with larger genetic distances observed within Africa [30]. Our findings thus suggest that, similarly to humans [31], tubercle bacilli emerged in Africa and then underwent early diversification followed by much more recent expansion of a successful clone to the rest of the world, possibly coinciding with the waves of human migration out of Africa. However, we cannot exclude the possibility that the geographical confinement of the smooth bacilli to Africa reflects failure to recognize smooth isolates found elsewhere as being genuine tubercle bacilli.

### Implications for Research

A longer interaction of tubercle bacilli with humans and the occurrence of recombination among tubercle bacilli have profound implications for debated questions such as the natural selection effect of tuberculosis on human populations, and the way tubercle bacilli have evolved their exceptional ability to persist for decades in host tissues [32–34]. These issues should be re-examined in the light of this new evolutionary perspective. Future studies will show whether the extensive sequence polymorphism observed in housekeeping genes goes hand in hand with nonsynonymous mutations in antigen-encoding genes or in genes encoding potential drug or diagnostic targets. Our findings may also have important consequences for strategies of research for immunoprotective and therapeutic targets, which until now have been based on the assumption of the intrinsically confined genetic variation of the pathogen restraining the possibilities of emergence of potential escape variants [7,35]. Comparative and functional genomic analyses of smooth tubercle bacilli, apparently confined to East Africa, and classical tubercle bacilli, found worldwide, will shed light on the selective advantages that led the latter to such a successful clonal expansion.

## Materials and Methods

### Mycobacterial isolates.

The tubercle bacilli isolates used in this study are listed in Table S1 (smooth isolates) and Table S3 (MTBC isolates). Most of the smooth tubercle isolates were recovered from African or European patients attending two French Military Medical Centres (Bouffard and Paul Faure) in Djibouti, East Africa. Three smooth isolates originally obtained by Georges Canetti and one smooth isolate obtained from Switzerland were included as references [36]. We also included type strains of each member of the MTBC as references.

### Distribution of repetitive DNA sequences and long sequence polymorphism markers*.*

Southern blots of genomic PvuII-digested tubercle bacilli DNA were sequentially probed with probes specific for IS*6110,* IS*1081,* DR region [17], region of difference *RD12^can^* [16], and *M. canettii* IS*Myca1* transposase. The probe specific for this transposase is a 650-bp DNA fragment obtained by using 5′-CAAGGTCAAGACGCGTACC-3′ and 5′-TGAGCTTGTCGATTTGAGCTT-3′ primers. PCR amplification of the fragments of IS*Myca1* flanking the transposase was perfomed using 5′-CTCGAACAGGTTCTGCTCATC-3′ and 5′-CGAAGTTCCCCCTTGTAGG-3′ primers. *RD12^can^* flanking regions were also amplified as previously described [16] and sequenced. To detect regions of difference *RD9* and *TbD1,* two PCR assays were done for each strain as previously described [16]. MIRU-VNTR analysis was performed via an automated technique using the target loci previously reported [37–40].

### DNA sequencing*.*

The whole 16S rRNA gene was amplified by using 5′-GCCGTTTGTTTTGTCAGGAT-3′ and 5′-GCTCGCAACCACTATCCAGT-3′ primers. The resulting product was sequenced using the following primers: 5′-GCCGTTTGTTTTGTCAGGAT-3′, 5′-CTGAGATACGGCCCAGACTC-3′, 5′-GCGCAGATATCAGGAGGAAC-3′, 5′-TCATGTTGCCAGCACGTAAT-3′, 5′-CCTACCGTCAATCCGAGAGA-3′, 5′-TGCATGTCAAACCCAGGTAA-3′, and 5′-TTCGGGTGTTACCGACTTTC-3′. To analyze polymorphisms in housekeeping genes, fragments of *katG, gyrA, gyrB, hsp65, rpoB,* and *sodA* genes were amplified and sequenced using previously published primers [7,41]. Each experiment was performed three times using different PCR products.

### Phylogenetic analyses.

Neighbor-joining trees were constructed using PAUP* version 4.0b10 with Jukes-Cantor distance correction (http://paup.csit.fsu.edu/). Trees were drawn using TreeView version 1.5 (http://taxonomy.zoology.gla.ac.uk/rod/treeview.html). Bootstrap analysis was performed with 1,000 replicates. Numbers of synonymous substitutions per synonymous site (Ks) and nonsynonymous substitutions per nonsynonymous site (Ka) were estimated using DNASP version 4.00, using the Nei and Gojobori method after Jukes-Cantor correction for multiple substitutions [42]. The program RDP version 2 [43] was used to detect mosaic sequences using the Sawyer's and chi-square methods. The RDP GENECONV algorithm (which looks for regions within a sequence alignment in which sequence pairs are sufficiently similar to suspect recombination) was used for Sawyer's test, with a *g*-scale parameter of one and using both sequence triplets or sequence pairs scanning methods. *p*-Values were obtained with the KA method. The chi-square method was implemented using the MaxChi algorithm of RDP. Given an alignment, MaxChi examines sequence pairs and seeks recombination breakpoints by comparing the number of variable and nonvariable sites on both sides of the breakpoint. Split decomposition analysis was performed using SplitsTree version 4b06 [24].

## Supporting Information

Figure S1 Genotypic Patterns of 37 Smooth Tubercle Bacilli Lanes 1 to 37 correspond to strains 1 to 37, respectively; line 38 corresponds to the reference strain *M. tuberculosis* Mt14323. Strains 1 and 6 are the reference strains *M. canettii* 140010059 and NZM 217/94, respectively; strains 8 and 17 are previously reported *M. canettii* strains (see Table S1). Lane groups A to I indicate the groups with identical genotypic patterns.
(A) DR region analysis by spoligotyping.
(B–E) Southern blot analysis with DNA probes against (B) the DR region, (C) IS*1081,* (D) IS*6110,* and (E) IS*Myca1,* a 1.8-kb insertion sequence related to the IS4 family (see Protocol S2).
(F) Southern blot analysis with a DNA probe directed against region *RD12^can^.* PCR using primers targeting the regions flanking *RD12^can^* and further sequencing of these amplification products demonstrated an identical deletion in groups A, C/D, E, and H, whereas deletion in group F overlapped *RD12^can^.*
(373 KB DOC)

Figure S2 Gene Phylogenies of *gyrA, gyrB, hsp65, katG,* and *rpoB* Sequences from the Eight Smooth Tubercle Bacilli Groups and the MTBC Members The unrooted trees were obtained using Megalign version 5.53 (DNASTAR, Madison, Wisconsin, United States).
(343 KB DOC)

Protocol S1 Estimation of Ks Value (25 KB DOC)

Protocol S2 IS*Myca1,* a New Insertion Sequence (27 KB DOC)

Table S1 Strains of Smooth Tubercle Bacilli (57 KB DOC)

Table S2 MIRU-VNTR Patterns of Smooth Tubercle Bacilli (361 KB DOC)

Table S3 MTBC Strains Used in This Study (26 KB DOC)

### Accession Numbers

The EMBL (http://www.ebi.ac.uk/embl/) accession numbers for the sequenced portions of *katG, gyrB, gyrA, rpoB,* and *hsp65* genes of the smooth tubercle bacilli are AJ749904–AJ749948. The *M. canettii* IS*Myca1* sequence has been deposited in the EMBL database under accession number AJ619854.

## Abbreviations

DR: direct repeat
MTBC: *Mycobacterium tuberculosis* complex
