# Supplementary material for: Ancient Origin and Gene Mosaicism of the Progenitor of Mycobacterium tuberculosis
Source: PLoS Pathog. 2005 Aug 19;1(1):e5. doi: 10.1371/journal.ppat.0010005 (PMC1238740; doi:10.1371/journal.ppat.0010005)

**Supporting Figure S1**

**Figure S1.** Genotypic patterns of 37 smooth tubercle bacilli. Lanes 1 to 37 correspond to strains 1 to 37 respectively, line 38 corresponds to the reference strain *M. tuberculosis* Mt14323. Strains 1 and 6 are the reference strains *M. canettii* 140010059 and NZM 217/94, respectively; strains 8 and 17 are previously reported *M. canettii* strains (Table S1). **A** to **I** indicate the groups with identical genotypic patterns. **a**, DR region analysis by spoligotyping. **b** to **e**, Southern blot analysis with DNA probes against (**b**) the DR region, (**c)** IS*1081*, (**d**) IS*6110*, and (**e)** IS*MycA1*, a 1.8-kb insertion sequence related to the IS4 family (Supporting Note 2). **f**, Southern blot analysis with a DNA probe directed against region RD12^can^. PCR using primers targeting the regions flanking RD12^can^ and further sequencing of these amplification products demonstrated an identical deletion in groups A, C/D, E and H, whereas deletion in group F overlapped RD12^can^.


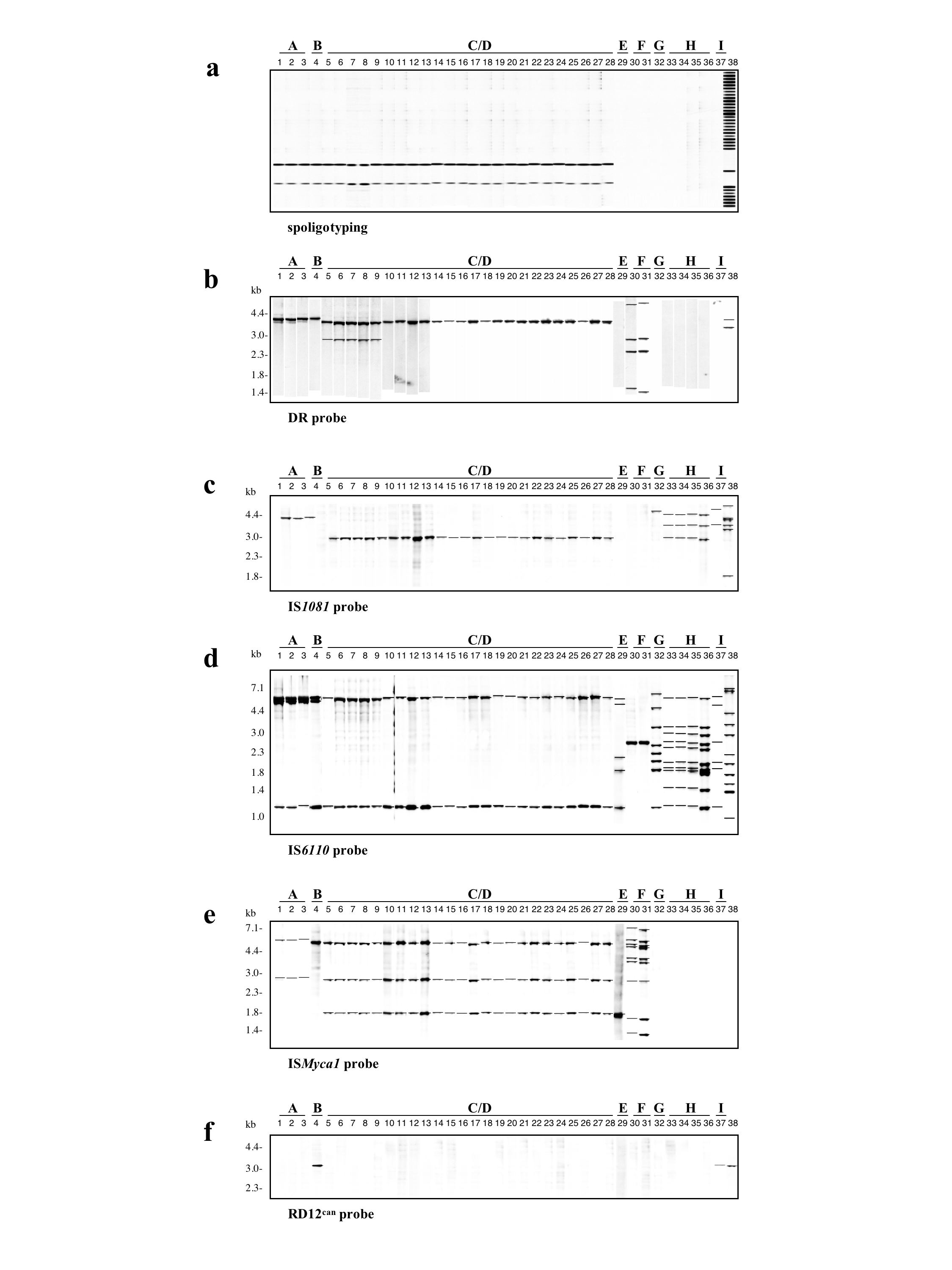

Supplement: Figure S1 — Lanes 1 to 37 correspond to strains 1 to 37, respectively; line 38 corresponds to the reference strain M. tuberculosis Mt14323. Strains 1 and 6 are the reference strains M. canettii 140010059 and NZM 217/94, respectively; strains 8 and 17 are previously reported M. canettii strains (see Table S1). Lane groups A to I indicate the groups with identical genotypic patterns. (A) DR region analysis by spoligotyping. (B–E) Southern blot analysis with DNA probes against (B) the DR region, (C) IS1081, (D) IS6110, and (E) ISMyca1, a 1.8-kb insertion sequence related to the IS4 family (see Protocol S2). (F) Southern blot analysis with a DNA probe directed against region RD12can. PCR using primers targeting the regions flanking RD12can and further sequencing of these amplification products demonstrated an identical deletion in groups A, C/D, E, and H, whereas deletion in group F overlapped RD12can. (373 KB DOC) [file ppat.0010005.sg001.doc]
