# Supplementary material for: Ancient Origin and Gene Mosaicism of the Progenitor of Mycobacterium tuberculosis
Source: PLoS Pathog. 2005 Aug 19;1(1):e5. doi: 10.1371/journal.ppat.0010005 (PMC1238740; doi:10.1371/journal.ppat.0010005)

**Supporting Figure S2**

Figure S2. Gene phylogenies of *gyrA*, *gyrB*, *hsp65*, *katG* and *rpoB* sequences from the eight smooth tubercle bacilli groups and the MTBC members. The unrooted trees were obtained using software Megalign v5.53 (DNASTAR Inc., Madison, WI).


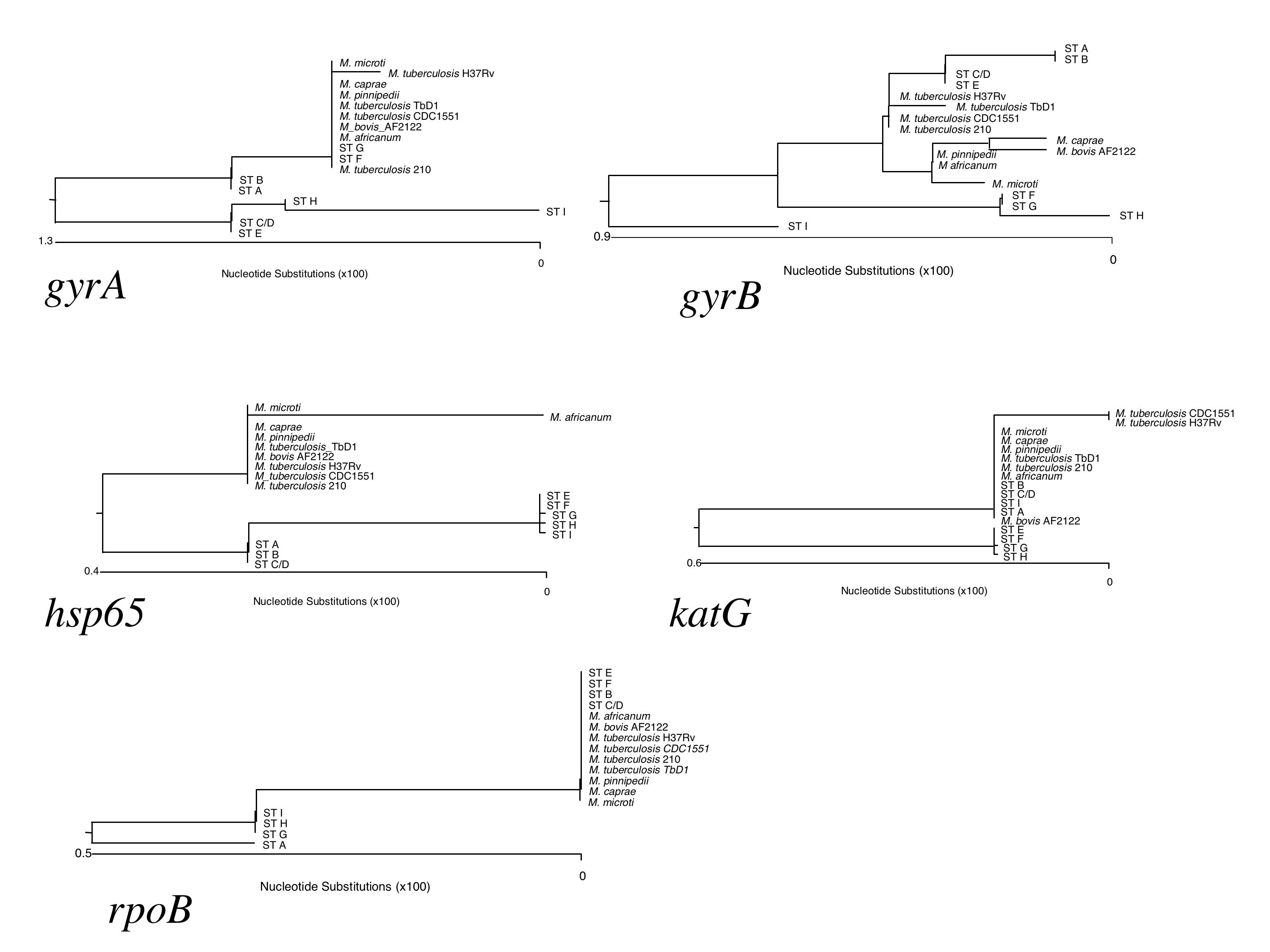

Supplement: Figure S2 — The unrooted trees were obtained using Megalign version 5.53 (DNASTAR, Madison, Wisconsin, United States). (343 KB DOC) [file ppat.0010005.sg002.doc]
