# Supplementary material for: Ancient Origin and Gene Mosaicism of the Progenitor of Mycobacterium tuberculosis
Source: PLoS Pathog. 2005 Aug 19;1(1):e5. doi: 10.1371/journal.ppat.0010005 (PMC1238740; doi:10.1371/journal.ppat.0010005)
Supplement: Protocol S1 — (25 KB DOC) [file ppat.0010005.sd001.doc]

**Supporting Note 1**

As we only sequenced representative strains from group C/D, this Ks value may be an overestimate. The minimal Ks value, obtained under the hypothesis that sequence variation is null within these groups, would be 0.0123. The rate of synonymous substitution used above is based on the assumption that *E. coli* and *Salmonella* diverged 100 million years ago[1]. If a higher rate of 0.03, estimated on the basis of *E. coli* laboratory mutation rate [2] were used, the estimated age would be 0.41 million years for the tubercle bacilli species and 5,000 years for MTBC.

#### References

1. Ochman H, Wilson AC (1987) Evolution in bacteria: evidence for a universal substitution rate in cellular genomes. J Mol Evol 26: 74-86.

2. Guttman DS, Dykhuizen DE (1994) Detecting selective sweeps in naturally occurring *Escherichia coli*. Genetics 138: 993-1003
