# Supplementary material for: Ancient Origin and Gene Mosaicism of the Progenitor of Mycobacterium tuberculosis
Source: PLoS Pathog. 2005 Aug 19;1(1):e5. doi: 10.1371/journal.ppat.0010005 (PMC1238740; doi:10.1371/journal.ppat.0010005)
Supplement: Protocol S2 — (27 KB DOC) [file ppat.0010005.sd002.doc]

### Supporting Note 2

IS*MycA1* (GenBank accession number AJ619854) was found in the genome of *M. canettii* and encodes a transposase that does not show any similarity with proteins from *M. tuberculosis* but shares 48 % amino acid sequence identity with transposases from IS elements carried by the *M. ulcerans* mega-plasmid [1]. Hybridization bands of strains 30 and 31 with IS*MycA1* probe correspond to an insertion sequence similar to IS*MycA1*, as demonstrated by PCR using primers targeting IS*Myca1*, which amplified inner fragments of the IS*Myca1* transposase but failed to amplify the terminal ones. The presence of these IS elements specific either to *M. canettii* or to other smooth groups is another significant distinctive character versus the MTBC.

**Reference**

1. Stinear TP, Mve-Obiang A, Small PL, Frigui W, Pryor MJ, et al. (2004) Giant plasmid-encoded polyketide synthases produce the macrolide toxin of *Mycobacterium ulcerans.* Proc Natl Acad Sci U S A 101: 1345-1349.
