# Supplementary material for: Ancient Origin and Gene Mosaicism of the Progenitor of Mycobacterium tuberculosis
Source: PLoS Pathog. 2005 Aug 19;1(1):e5. doi: 10.1371/journal.ppat.0010005 (PMC1238740; doi:10.1371/journal.ppat.0010005)
Supplement: Table S1 — (57 KB DOC) [file ppat.0010005.st001.doc]

**Supporting table S1**

# Table S1. Strains of smooth tubercle bacilli

| Strain number | Strain code | Site of tuberculosis | Year of isolation | Country of isolation | Genetic  pattern |
| --- | --- | --- | --- | --- | --- |
| 1^a^ | 140010059 | pulmonary | 1969 | France | A |
| 2^a^ | 140010060 | pulmonary | 1969 | France | A |
| 3^a^ | 140010061 | pulmonary | 1970 | Papeete | A |
| 4 | 19990711 | lymph node | 1999 | Djibouti | B |
| 5 | 19970130 | pulmonary | 1997 | France | C |
| 6^b^ | NZM 217/94 | lymph node | 1993 | Switzerland | C |
| 7 | 19990161 | lymph node | 1999 | Djibouti | C |
| 8^c,d^ | 19990160 | pulmonary | 1999 | France | C |
| 9 | 19981514 | lymph node | 1998 | Djibouti | C |
| 10 | 19980862 | lymph node | 1998 | Djibouti | D |
| 11 | 19990516 | pulmonary | 1999 | Djibouti | D |
| 12 | 19990515 | pulmonary | 1999 | Djibouti | D |
| 13 | 19991704 | pulmonary | 1999 | Djibouti | D |
| 14 | 20000342 | NA | 2000 | Djibouti | D |
| 15 | 19991708 | lymph node | 1999 | Djibouti | D |
| 16^c^ | 20000586 | pulmonary | 2000 | Djibouti | D |
| 17^c,d^ | 19991574 | pulmonary | 1999 | France | D |
| 18 | 19990589 | pulmonary | 1999 | Djibouti | D |
| 19^c^ | 20001155 | pulmonary | 2000 | France | D |
| 20 | 20001246 | pulmonary | 2000 | Djibouti | D |
| 21 | 20001245 | pulmonary | 2000 | Djibouti | D |
| 22 | 20010188 | lymph node | 2001 | Djibouti | D |
| 23 | 20001248 | pulmonary | 2000 | Djibouti | D |
| 24 | 20001247 | peritoneal liq. | 2000 | Djibouti | D |
| 25 | 20010390 | lymph node | 2001 | Djibouti | D |
| 26 | 20010389 | peritoneal liq. | 2001 | Djibouti | D |
| 27 | 20010933 | lymph node | 2001 | Djibouti | D |
| 28 | 20001049 | pulmonary | 2001 | Djibouti | D |
| 29 | 19991709 | lymph node | 1999 | Djibouti | E |
| 30^e^ | 19990263 | pulmonary | 1997 | France | F |
| 31 | 20010391 | pulmonary | 2001 | Djibouti | F |
| 32 | 20000587 | lymph node | 2000 | Djibouti | G |
| 33 | 19980865 | pulmonary | 1998 | Djibouti | H |
| 34 | 19980864 | pulmonary | 1998 | Djibouti | H |
| 35 | 19980863 | pulmonary | 1998 | Djibouti | H |
| 36 | 19991705 | pulmonary | 1999 | Djibouti | H |
| 37 | 20000473 | pulmonary | 2000 | Djibouti | I |

^a^ *M. canettii* from the CNRM collection (Institut Pasteur) originally isolated by Georges Canetti. No epidemiological data are available for these patients.

^b^ *M. canettii* NZM 217/94 kindly provided by G. Pfyffer (Swiss National Reference Center for Mycobacteria, Zurich, Switzerland). This strain was isolated from a Swiss patient who lived in Uganda for 20 years and in Kenya for 1 year.

^c^ French patient who lived in, or living in, Djibouti.

^d^ *M. canettii* previously reported by Miltgen et al [1].

^e^ Smooth tubercle bacilli kindly provided by L. Desforges (Hop. Henri Mondor, Paris, France). The strain was isolated from a Djibutian patient living in France.

NA, not available.

**Reference**

1. Miltgen J, Morillon M, Koeck JL, Varnerot A, Briant JF, et al. (2002) Two cases of pulmonary tuberculosis caused by *Mycobacterium tuberculosis* subsp canetti. Emerg Infect Dis 8: 1350-1352.
