# Supplementary figures and images for: Ancient Origin and Gene Mosaicism of the Progenitor of Mycobacterium tuberculosis
Source: PLoS Pathog. 2005 Aug 19;1(1):e5. doi: 10.1371/journal.ppat.0010005 (PMC1238740; doi:10.1371/journal.ppat.0010005)

#### Supporting Table S2

## Table S2. MIRU-VNTR patterns of smooth tubercle bacilli


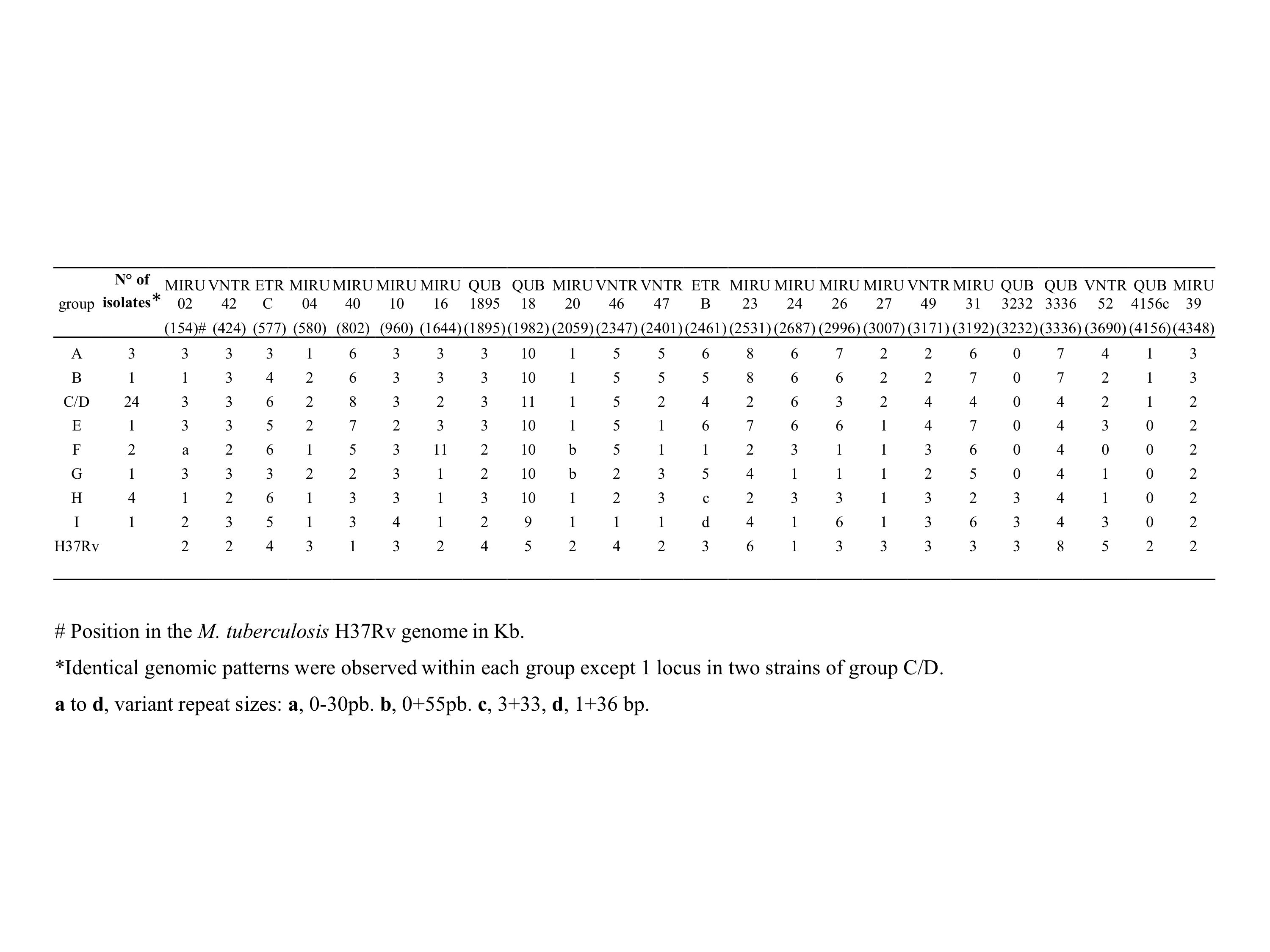

Supplement: Table S2 — (361 KB DOC) [file ppat.0010005.st002.doc]
