# Supplementary material for: Ancient Origin and Gene Mosaicism of the Progenitor of Mycobacterium tuberculosis
Source: PLoS Pathog. 2005 Aug 19;1(1):e5. doi: 10.1371/journal.ppat.0010005 (PMC1238740; doi:10.1371/journal.ppat.0010005)
Supplement: Table S3 — (26 KB DOC) [file ppat.0010005.st003.doc]

Supporting Table S3

**Table S3**. MTBC strains used in this study

|  | PGG* | TbD1^#^ | |
| --- | --- | --- | --- |
| *M. tuberculosis*  CIPTB° 20030423  210 (W)  CDC1551  H37Rv (type strain) | 1  1  2  3 | +  -  -  - |  |
| *M. africanum* CIPTB 140030001 (type strain) | 1 | + |  |
| *M. bovis* AF122/97 (type strain) | 1 | + |  |
| *M. microti* CIPTB 140050001 (type strain) | 1 | + |  |
| *M. pinnipedii* CIPTB 140090001 | 1 | + |  |
| *M. caprae* CIPTB 140080012 (type strain) | 1 | + |  |

*Principal Genetic Group as defined by Sreevatsan, S. et al.

^#^ Presence (+) or absence (-) of the region of difference TbD1.

° CIPTB : Collection Institut Pasteur, Tuberculose.
